# Optimization of a Bayesian penalized likelihood algorithm (Q.Clear) for ^18^F-NaF bone PET/CT images acquired over shorter durations using a custom-designed phantom

**DOI:** 10.1186/s40658-020-00325-8

**Published:** 2020-09-11

**Authors:** Tokiya Yoshii, Kenta Miwa, Masashi Yamaguchi, Kai Shimada, Kei Wagatsuma, Tensho Yamao, Yuto Kamitaka, Seiya Hiratsuka, Rinya Kobayashi, Hajime Ichikawa, Noriaki Miyaji, Tsuyoshi Miyazaki, Kenji Ishii

**Affiliations:** 1 Department of Radiological Sciences, School of Health Science, International University of Health and Welfare, 2600-1 Kitakanemaru, Ohtawara, Tochigi, 324-8501 Japan; 2 Department of Radiology, Fukushima Medical University Hospital, 1 Hikarigaoka, Fukushima, Fukushima 960-1247 Japan; 3 Research Team for Neuroimaging, Tokyo Metropolitan Institute of Gerontology, 35-2, Sakae-cho, Itabashi-ku, Tokyo, 173-0015 Japan; 4 Department of Radiology, Toyohashi Municipal Hospital, 50, Aza Hachiken Nishi, Aotake-Cho, Toyohashi, Aichi 441-8570 Japan; 5 Department of Nuclear Medicine, Cancer Institute Hospital of Japanese Foundation for Cancer Research, 3-8-31 Ariake, Koto-ku, Tokyo, 135-8550 Japan; 6 Department of Orthopaedic Surgery, Tokyo Metropolitan Geriatric Hospital and Institute of Gerontology, 35-2, Sakae-cho, Itabashi-ku, Tokyo, 173-0015 Japan

**Keywords:** Q.Clear, Quantitation, ^18^F-NaF, SiPM, BPL, TOF

## Abstract

**Background:**

The Bayesian penalized likelihood (BPL) algorithm Q.Clear (GE Healthcare) allows fully convergent iterative reconstruction that results in better image quality and quantitative accuracy, while limiting image noise. The present study aimed to optimize BPL reconstruction parameters for ^18^F-NaF PET/CT images and to determine the feasibility of ^18^F-NaF PET/CT image acquisition over shorter durations in clinical practice.

**Methods:**

A custom-designed thoracic spine phantom consisting of several inserts, soft tissue, normal spine, and metastatic bone tumor, was scanned using a Discovery MI PET/CT scanner (GE Healthcare). The phantom allows optional adjustment of activity distribution, tumor size, and attenuation. We reconstructed PET images using OSEM + PSF + TOF (2 iterations, 17 subsets, and a 4-mm Gaussian filter), BPL + TOF (β = 200 to 700), and scan durations of 30–120 s. Signal-to-noise ratios (SNR), contrast, and coefficients of variance (CV) as image quality indicators were calculated, whereas the quantitative measures were recovery coefficients (RC) and RC linearity over a range of activity. We retrospectively analyzed images from five persons without bone metastases (male, *n* = 1; female, *n* = 4), then standardized uptake values (SUV), CV, and SNR at the 4th, 5th, and 6th thoracic vertebra were calculated in BPL + TOF (β = 400) images.

**Results:**

The optimal reconstruction parameter of the BPL was β = 400 when images were acquired at 120 s/bed. At 90 s/bed, the BPL with a β value of 400 yielded 24% and 18% higher SNR and contrast, respectively, than OSEM (2 iterations; 120 s acquisitions). The BPL was superior to OSEM in terms of RC and the RC linearity over a range of activity, regardless of scan duration. The SUV_max_ were lower in BPL, than in OSEM. The CV and vertebral SNR in BPL were superior to those in OSEM.

**Conclusions:**

The optimal reconstruction parameters of ^18^F-NaF PET/CT images acquired over different durations were determined. The BPL can reduce PET acquisition to 90 s/bed in ^18^F-NaF PET/CT imaging. Our results suggest that BPL (β = 400) on SiPM-based TOF PET/CT scanner maintained high image quality and quantitative accuracy even for shorter acquisition durations.

## Introduction

Positron emission tomography/computed tomography (PET/CT) with ^18^F-sodium fluoride (^18^F-NaF) is clinically applied to detect bone metastases derived from a wide range of primary tumors [1–3]. ^18^F-NaF PET/CT is more sensitive, specific, and diagnostically accurate than traditional bone planar imaging and single-photon emission computed tomography (SPECT) using ^99m^Tc-labeled phosphate compounds [4, 5]. Responses to therapy for bone metastases can also be assessed by ^18^F-NaF PET/CT using quantitative indices such as standardized uptake values (SUV) [6]. However, prolonged acquisition > 30 min can be uncomfortable for patients with bone metastatic pain, and risk of patient motion is increased; thus, more rapid ^18^F-NaF PET/CT image acquisition is needed [7, 8].

The Society of Nuclear Medicine and Molecular Imaging (SNMMI) and the European Association of Nuclear Medicine (EANM) [4, 9] practice guidelines for ^18^F-NaF PET/CT imaging recommend that the ^18^F-NaF PET/CT tumor imaging protocol should be identical to that of ^18^F-fluoro-2-deoxy-D-glucose (^18^F-FDG) PET/CT. However, optimum image acquisition and reconstruction parameters in terms of ^18^F-NaF PET/CT imaging have not been described in detail. The reconstruction parameters of ^18^F-NaF PET/CT should be optimized for rapid image acquisition because image quality and quantitative accuracy depend on the amount and type of injected radiotracer activity and image reconstruction.

The software for image reconstruction and the hardware (detector material and design) have been upgraded in contemporary PET systems to improve image quality and quantitation [10, 11]. The Bayesian penalized likelihood reconstruction (BPL) algorithm, Q.Clear® (GE Healthcare, Waukesha, WI, USA), has recently been clinically applied. The BPL runs to full convergence of image accuracy while suppressing image noise using a penalty function. It also includes point spread function (PSF) modeling and controls image noise through a penalization factor (β value), which determines the global strength of regularization [12]. Compared with conventional ordered-subset expectation maximization (OSEM) reconstruction, BPL offers a higher signal-to-noise ratio (SNR) and more accurate quantitation over shorter acquisition durations [13]. Silicon photomultipliers (SiPMs) have recently replaced photomultiplier tubes (PMTs) which have led to PET detectors with smaller crystals, better timing resolution, and higher photon-detection efficiency [10, 14]. GE Healthcare introduced the first SiPM-based PET/CT scanner (Discovery MI; DMI; GE Healthcare), and it has delivered better sensitivity and high time-of-flight (TOF) performance gain. The higher sensitivity and peak noise equivalent count rate (NECR) of the DMI delivered the same SNR within ~ 40% shorter acquisition duration, compared with conventional PMT-based PET/CT scanners such as the Discovery 690 (GE Healthcare) [11].

The optimal β value in BPL should be determined by balancing contrast recovery and image noise [13]. Lindström et al. concluded that a β value of 400 for ^18^F-FDG whole body scans would be optimal when using BPL on a SiPM-based TOF PET/CT scanner [15]. However, acquisitions over a 3-min/bed position applied in their study can be clinically problematic for total-body ^18^F-NaF PET/CT image acquisition from the vertex to the toes in terms of patient comfort and throughput [16]. De Bernardi et al. used a regularized reconstruction similar to BPL to reduce the acquisition duration by about one-third [7], whereas Sonni et al. stated that DMI reduced PET imaging acquisition to 90 s/bed [17]. Furthermore, Lindström et al. showed that the acquisition durations could be reduced from 3 to 2 min/bed when BPL was used instead of OSEM on the DMI scanner [15]. Therefore, we postulated that BPL on the SiPM-based PET/CT scanner can reduce the amount of time required for ^18^F-NaF PET/CT acquisition, while maintaining image quality and quantitative accuracy. The present study aimed to optimize the image reconstruction parameters of BPL in ^18^F-NaF PET/CT imaging using a custom-designed phantom simulating a patient with bone metastases and to determine the feasibility of decreasing the duration of ^18^F-NaF PET/CT image acquisition in clinical practice.

## Materials and methods

### PET/CT scanner

All PET data were acquired using the DMI PET/CT system with a PET scanner comprising four rings of detector blocks with LYSO crystals coupled to a SiPM array. The LYSO scintillator (LightBurst digital detector) unit includes 19,584 LYSO 3.95 × 5.3 × 25-mm crystals in a 4 × 9 matrix. The scanner has 36 detector units per ring and 9792 SiPM channels. The PET detector has axial and transaxial fields of view (FOV) of 20 and 70 cm, respectively. The timing resolution is 375 ps. The spatial resolution, sensitivity, and observed peak NECR of the scanner according to NEMA NU 2-2007 is 3.91 mm in full width at half maximum (FWHM) at 10 mm off center, 12.62 cps/kBq, and 185.6 kcps at 22.5 kBq/mL, respectively [10]. The PET system is combined with a 64-slice CT. The CT data are used for attenuation correction.

### Phantom design

**Fig. 1:**
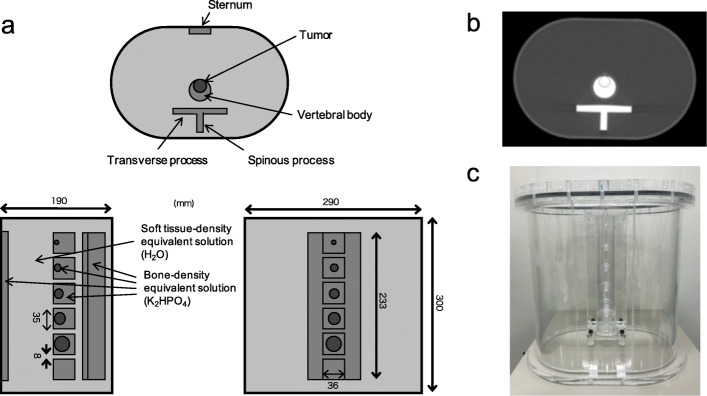
Custom-made thoracic spine phantom. Simplified schema (**a**), CT image (**b**), and photograph (**c**) of phantom. Example of setup shows vertebral body phantoms with tumors of 10, 13, 17, 22, and 28 mm in diameter. Vertebral body phantom without tumors is at the bottle. Vertebral body, tumor, processus, and sternum contain K_2_HPO_4_ solution with density equivalent to that of bone and ^18^F-NaF. Elliptical body phantom contained ^18^F-NaF

A custom-designed thoracic spine phantom (Fig. 1) comprised the trunk of a body phantom with a sternum, soft tissue, normal spine, and simulated bone tumors with diameters of 10, 13, 17, 22, and 28 mm. The main body phantom was elliptical with major and minor axes of 290 and 190 mm, respectively, and a height of 300 mm, which simulated a standard Japanese person weighing 60 kg. The vertebral body, tumor, and normal spine of the phantom contained a solution of dipotassium hydrogen phosphate (K_2_HPO_4_) with a density equivalent to that of bone. When K_2_HPO_4_ (100 g) is dissolved in 67 g of water as suggested by de Dreuille et al. [18], the composition of the K_2_HPO_4_ solution is 26% (K), 10% (P), 56% (O), and 8% (H) which is comparable to that of cranial bone: 17.6% (Ca), 8.1% (P), 43.5% (O), 5% (H), 21.2% (C), and 4% (N). The density of this K_2_HPO_4_ solution was 1.68 g/cm^3^ which is close to that of bone (1.61 g/cm^3^) [18]. The phantom allows optional adjustment of the activity distribution, tumor size, and linear attenuation coefficient (cm^−1^); thus, scatter and photon attenuation due to bone are considered [19]. A previous study found that the attenuation coefficient of K_2_HPO_4_ solution for 511 keV photons was 0.206 cm^−1^ [20].

All phantom experiments were conducted twice using spheres with different diameters and activity concentrations as follows. In the first round of experiments, the phantom consisted of spheres with diameters of 10, 13, 17, 22, and 28 mm containing a solution of ^18^F-NaF. The activity concentrations (AC) in the soft tissue, normal spine, and simulated tumor were 2.6, 15.6, and 62.4 kBq/mL, respectively, that is, tumor-to-normal bone ratio (TNR) of 4 [21–23]. In the second round of experiments, five spheres with diameters of 13 mm were set at TNR of 1, 2, 4, 8, and 16 at the normal spine activity concentration of 15.6 kBq/mL [24].

### Data acquisition and image reconstruction

All emission data were acquired in three-dimensional (3D) list mode. Subsequently, PET images acquired from 30 to 120 (30, 45, 60, 90, and 120) s/bed were reconstructed using OSEM + PSF + TOF (VPFX-S) and BPL + TOF (Q.Clear + TOF, QCFX-S). We applied two iterations, 17 subsets and a 4-mm Gaussian filter to OSEM, whereas the β value in BPL varied from 200 to 700 at intervals of 100. The FOV was 50 cm and the matrix was 256 × 256 (pixel size 1.95 × 1.95 mm^2^, slice thickness 2.79 mm).

### Data analysis

Phantom PET images were analyzed using OsiriX MD software ver.10.0.5 (Pixmeo Sàrl, Bernex, Switzerland). We placed 80% circular regions of interest (ROI) on a slice of the center tumor region, a slice of the normal bone center, and slices ± 1 and ± 2 slices from the central slice. We then calculated the SNR, contrast of a 10- or 13-mm hot sphere, and coefficients of variance (CV) as indicators of image quality. The SNR_10 and 13 mm_ was calculated as [25] follows:

$$ {\mathrm{SNR}}_{10\ \mathrm{and}\ 13\ \mathrm{mm}}=\frac{{\mathrm{AC}}_{10\ \mathrm{and}\ 13\ \mathrm{mm}\ \mathrm{hot}\_\max }-{\mathrm{AC}}_{\mathrm{bone}\_\mathrm{mean}}}{{\mathrm{SD}}_{\mathrm{bone}}}, $$

where AC_10 and 13 mm hot_max_ was the maximum measured activity concentration in the 10- and 13-mm hot sphere ROI, AC_bone_mean_ was the mean measured activity concentration in the normal bone ROI, and SD_bone_ was the standard deviation of the activity concentration in normal bone ROIs. The contrast of the 10-mm hot sphere and CV were respectively calculated as [25] follows:

$$ \mathrm{Contrast}=\kern0.5em \frac{{\mathrm{AC}}_{10\ \mathrm{mm}\ \mathrm{hot}\_\max }\ }{{\mathrm{AC}}_{\mathrm{bone}\_\mathrm{mean}}} $$

and

$$ \mathrm{CV}=\frac{\ {\mathrm{SD}}_{\mathrm{bone}}\ }{{\mathrm{AC}}_{\mathrm{bone}\_\mathrm{mean}}}, $$

We calculated the absolute recovery coefficients (RC) and the RC linearity over a range of activity as indicators of quantitation. The RC was calculated as follows:

$$ \mathrm{RC}=\kern0.5em \frac{{\mathrm{AC}}_{\mathrm{hot}\_\mathrm{mean}}}{\mathrm{True}\ \mathrm{AC}}, $$

where AC_hot_mean_ was the mean measured activity concentration in each sphere. The True AC was measured using a BeWell Model-QS03 F/B well counter (Molecular Imaging Lab, Suita, Japan) and considered as a reference. The absolute error of the well counter determined using a National Institute of Standards and Technology (NIST)-traceable, rod-shaped ^68^Ge/^68^Ga source was − 0.77% in this study [26]. The PET scanner was cross-calibrated to the dose calibrator or the well counter using ^18^F solution according to the vendor’s recommended procedures. The RC linearity over a range of activity for OSEM and BPL was evaluated as the relationship between AC_hot_mean_ and True AC*.*

### Clinical study

The present study proceeded in accordance with the Declaration of Helsinki, and was approved by the Ethics Committee at the TMIG (Approval Nos. 250413 and 28077). All applicants provided written informed consent to participate in the study after physicians explained the study in detail. We acquired images from five persons (male, *n* = 1; female, *n* = 4; median age, 81 years; range, 77–85; average weight, 58.8 ± 9.9 kg; 52–68 kg) using DMI PET/CT scanners (GE Healthcare). The absence of bone metastases on ^18^F-NaF PET images was confirmed in all of them. We acquired PET/CT images at 45 min after injecting an average of 232.8 ± 34.8 (192–258) MBq of ^18^F-NaF. The scan duration per bed position (determined in phantom studies) was 90 s, and patients were scanned in 13 or 14 bed positions. All PET images were reconstructed under the following conditions: OSEM + PSF + TOF (2 iterations, 17 subsets, and a 4-mm Gaussian filter) and BPL + TOF (β = 400). Optimal reconstruction parameters were determined from phantom studies.

The quantitative performance and noise characteristics of the clinical PET image were analyzed at the level of the 4th, 5th, and 6th thoracic vertebrae. We adjusted and placed a sphere ROI of 80% size on the center of the axial slice in the section after measuring the ROI of the vertebral body guided by the CT boundaries of the fused PET/CT images. The mean and maximum standardized uptake values (SUV_mean_ and SUV_max_, respectively), CV and vertebral SNR of target thoracic vertebrae were calculated. The CV was defined as the standard deviation (SD) normalized to the SUV_mean_ of the ROI placed in the vertebral body. The vertebral SNR was calculated as SUV_mean_ in the vertebral body divided by the CV. The data were analyzed using PETSTAT software (AdIn Research, Tokyo, Japan).

## Results

**Fig. 2:**
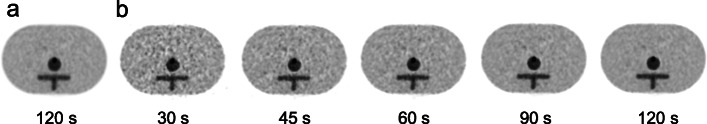
Sample PET images acquired from a 13-mm sphere with different acquisition durations and reconstructed using OSEM (2 iterations) (**a**) and BPL (β value, 400) (**b**)

Figure 2 shows representative axial images acquired over various acquisition durations in the phantom study. A shorter acquisition time caused increased background noise. The 13-mm hot sphere was clearly recognized in BPL with an acquisition duration of ≥ 90 s, which was comparable to that in OSEM with 120 s. The SNRs of a 13-mm hot sphere (SNR_13mm_) in BPL with 90 s and OSEM with 120 s were 35.5 and 35.3, respectively.

**Fig. 3:**
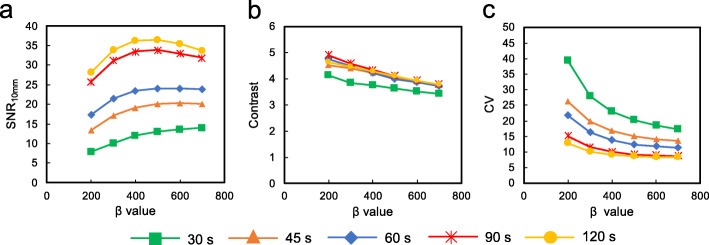
PET image quality of 10-mm spheres as a function of β value (range 200–700) for each acquisition duration using BPL. **a** SNR_10mm_. **b** Contrast. **c** CV

Figure 3 shows the SNR_10 mm_, contrast, and CV of 10-mm spheres as a function of the β value for each duration. The SNR_10 mm_ was maximal at β values of 400–500 and subsequently decreased as β values increased. As the β value increased, the contrast and CV decreased. The SNR_10 mm_ increased with increasing β values from 200 to 400 or 700 depending on the acquisition duration. The SNR_10mm_ and noise characteristics also improved with increasing acquisition duration for a given β value. The contrast was independent of acquisition duration at ≥ 45 s/bed.

**Fig. 4:**
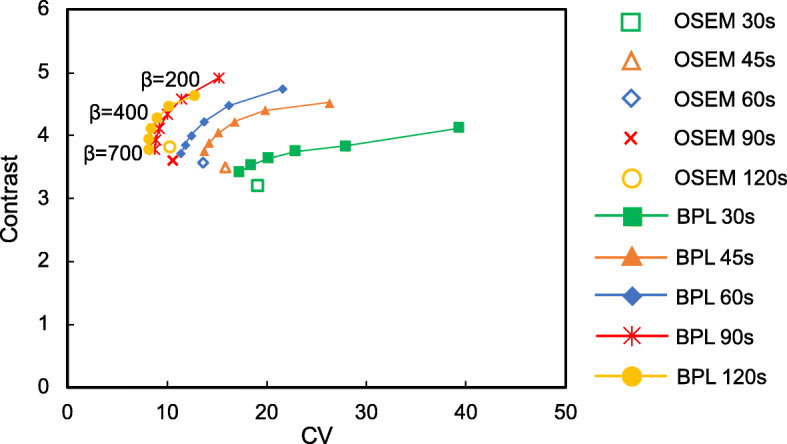
Relationship between contrast and CV curves of hot spheres with 10-mm diameter using OSEM and BPL reconstructions after each acquisition duration. Plots of OSEM correspond to 2 iterations. Curves for BPL run from left to right with decreasing β values, respectively. Unfilled and filled symbols represent OSEM and BPL, respectively

Figure 4 shows the relationship between contrast and CV for OSEM and BPL. Contrast is plotted as a function of the CV for hot spheres with diameters of 10 mm, and each plot corresponds to iterations of 2 and β values ranging from 200 to 700 in OSEM and BPL, respectively. As the value of β increased, contrast and CV decreased during all acquisitions. Therefore, a choice is needed between increased contrast and decreased CV. Ideally these points on lie in the top left of the graph [12]. The balance between contrast and CV was optimal at a β value of 400 for BPL. The contrast of BPL was superior to that of OSEM at comparable noise levels. Based on these results, we recommend a β value of 400 for BPL. The BPL (β value, 400) during 90-s acquisitions yielded similar noise levels to those obtained with OSEM (2 iterations) during 120-s acquisitions. The BPL with a β value of 400 improved SNR and contrast by 24% and 18%, respectively, compared with OSEM.

**Fig. 5:**
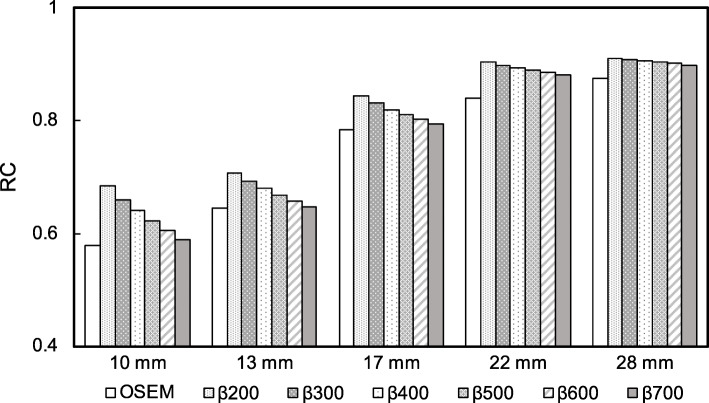
Recovery coefficient as a function of sphere size with different methods of reconstructing images acquired over 120 s

Figure 5 shows the RC for OSEM (2 iterations) and BPL (β values, 200–700) for each sphere size. The RC for OSEM decreased at smaller sphere diameter. The RC for BPL were superior to those for OSEM. The RCs decreased with increasing β values for all sphere sizes, just more significantly for the smaller sphere sizes.

**Fig. 6:**
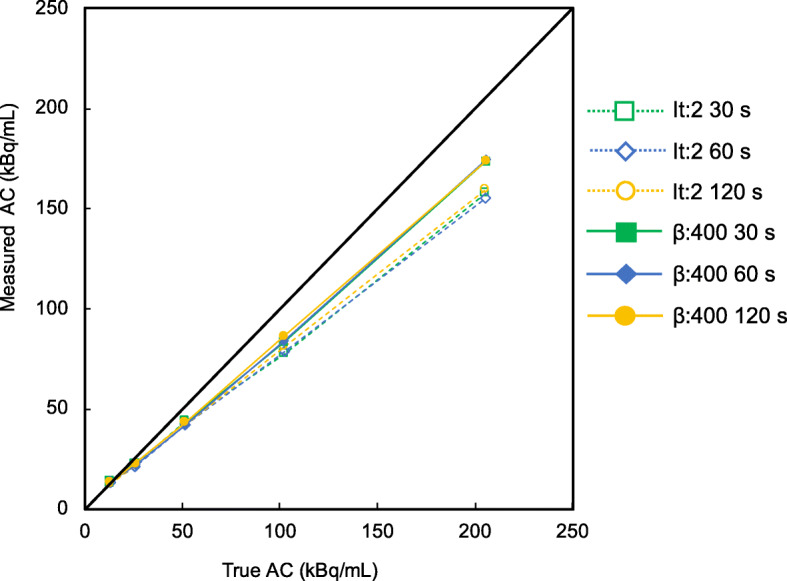
Correlation between true and measured activity concentrations. The RC linearity over a range of activity was measured using five spheres with diameters of 13 mm containing TNR of 1, 2, 4, 8, and 16 at the normal spine activity concentration of 15.6 kBq/mL. Unfilled symbols and dotted lines, OSEM; filled symbols and solid line, BPL. AC, activity concentration

Figure 6 shows the RC linearity over a range of activity for OSEM (iteration, 2) and BPL (β values, 400) of images acquired during different acquisition durations. The BPL was more linear than OSEM because values measured using BPL were closer to the true values. Linearity for OSEM slightly varied and was better for images acquired over longer durations, whereas BPL was independent of the acquisition duration.

**Fig. 7:**
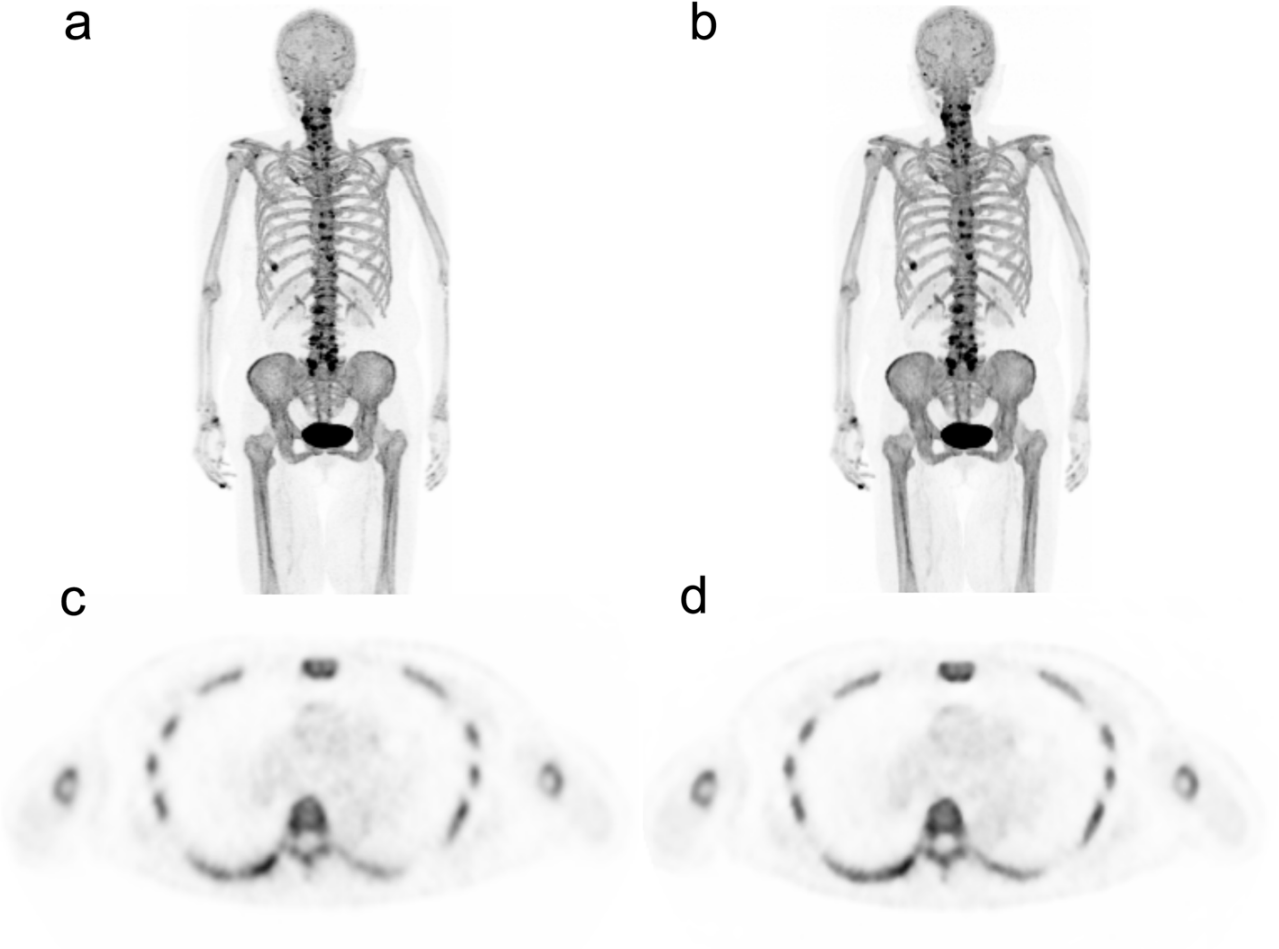
^18^F-NaF PET images of a 77-year-old female (weight 63 kg). Maximum intensity projection (MIP) and axial PET images reconstructed by **a**, **c** OSEM (2 iterations) and **b**, **d** BPL (β value, 400), respectively. The hotspots of osteoblastic activity in MIP images (**a**, **b**) show the degenerative bone changes and bone bruises

**Fig. 8:**
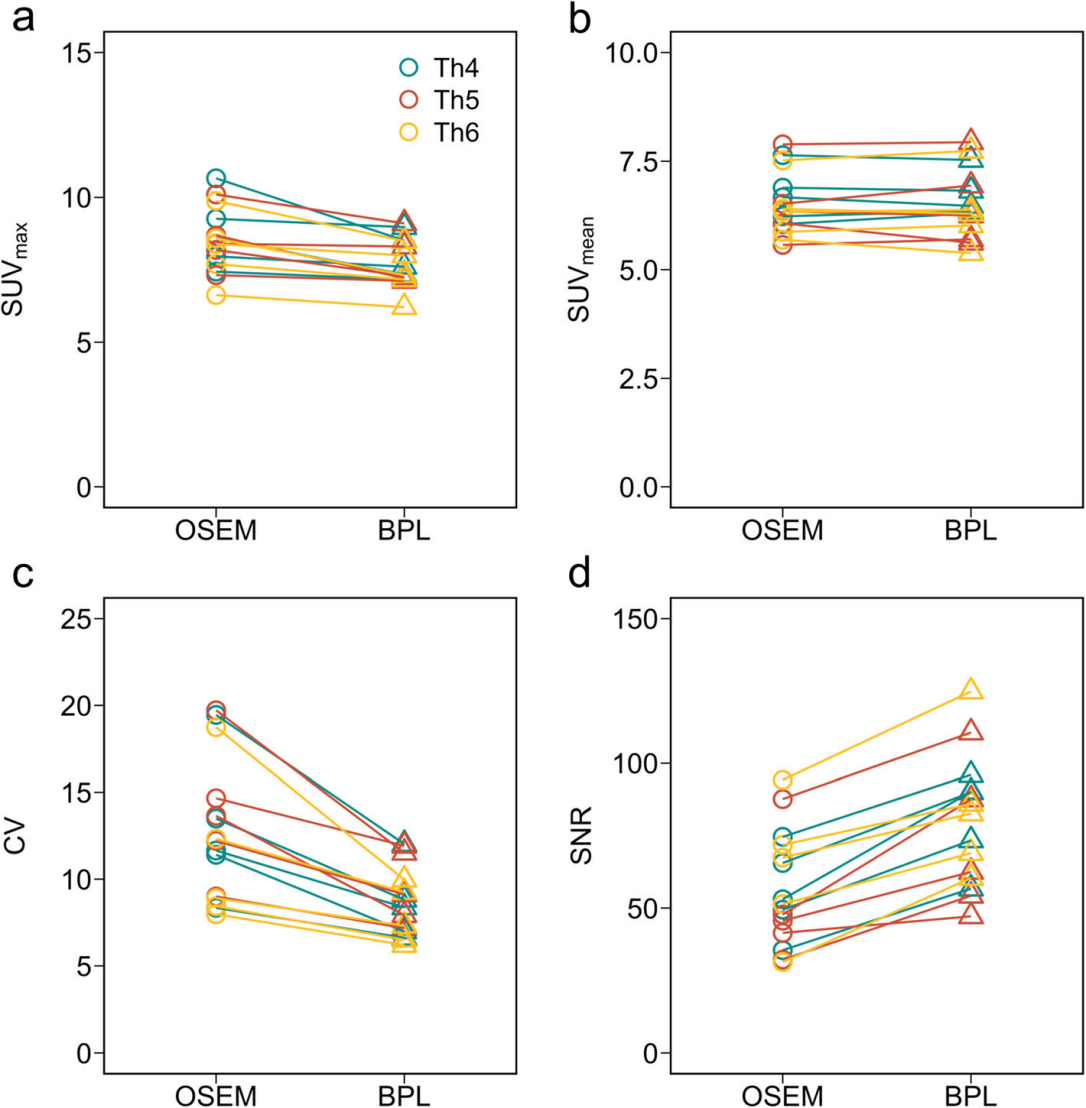
Clinical comparisons of **a** SUV_max_, **b** SUV_mean_, **c** CV, and **d** vertebral SNR under optimal reconstruction conditions (OSEM, 2 iterations; BPL, β value, 400)

Figure 7 shows whole-body maximum intensity projection (MIP) and axial PET images acquired using DMI with OSEM and BPL reconstruction in the clinical study. The axial PET images exhibited slightly more uptake edge preservation and noise suppression in the vertebral body when reconstructed using BPL. Figure 8 shows the results of SUV_max_, SUV_mean_, CV, and vertebral SNR of five persons under optimal reconstruction conditions (OSEM: iterations, 2; BPL: β value, 400). The SUV_max_ were lower in BPL than in OSEM. The noise characteristics and vertebral SNR in BPL were superior to those of OSEM.

## Discussion

We evaluated the image quality and quantitative accuracy of BPL for reconstructing images acquired using a SiPM-based PET/CT scanner and a custom-designed phantom containing ^18^F-NaF and a bone-equivalent solution of K_2_HPO_4_. We found that BPL on the DMI improved SNR_10mm_, contrast, noise characteristics, and quantitation compared to OSEM, even though the images were acquired over a duration of 90, instead of 120 s.

The optimum image acquisition and reconstruction parameters of ^18^F-FDG and ^18^F-NaF PET/CT imaging has been considered to be identical [4, 9]. However, biodistribution and lesion uptake levels of ^18^F-FDG and ^18^F-NaF are significantly different [27]. Optimal reconstruction parameters depend on the radiotracer biodistribution and lesion uptake levels [28]. Although the parameters of BPL have been optimized for various radiotracers [29–31], ^18^F-NaF PET/CT has not yet been evaluated. The activity concentration and linear attenuation coefficient (cm^−1^) can be modified in three different locations of soft tissue, normal spine, and simulated tumor in bone using our custom-designed phantom. Thus, the effects of scatter and attenuation of photons by bone for ^18^F-NaF PET/CT imaging can be investigated [19].

Contrast and image noise in PET images decreased as the β value increased [12, 32, 33]. The highest SNR_10 mm_ were at β values of 400–500. A trade-off needs to be reached between the two conflicting performance parameters of high contrast and low image noise for determining an optimal β value [25, 32]. The balance of contrast and CV was optimal when a β value was 400 in the present study, in which the β value was independent of the acquisition duration at ≥ 45 s (Fig. 4). This result was comparable with previous findings of oncologic PET imaging using ^18^F-fluciclovine (300) [29], ^68^Ga-prostate-specific membrane antigen (400–550) [31], and ^18^F-FDG (350–400) [12, 15, 32]. Figure 4 shows that BPL achieved higher contrast and lower image noise than OSEM. The PET images for BPL were superior to those for OSEM with the optimal reconstruction parameters (2 iterations; 120 s acquisitions), even though acquisition was reduced from 120 to 90 s. This is in agreement with the studies of De Bernardi et al. [7] and Lindström et al. [15] who found that regularized reconstruction can result in reduced acquisition durations. We therefore believe that BPL can reduce PET acquisition to 90 s/bed in ^18^F-NaF PET/CT imaging.

The SiPM photodetectors in the DMI PET/CT scanner are characterized by excellent intrinsic timing resolution (375 ps in the DMI) and higher sensitivity [11]. The spatial localization along a line of response calculated from Δ*x* = *c* × Δ*t*/2 (Δ*x*, spatial localization; *c*, light speed; Δ*t*, timing resolution) is 5.8 cm in the DMI [34]. The TOF of DMI should obtain a good SNR even during shorter acquisitions. Moreover, sensitivity was better for DMI PET/CT than PMT-based TOF PET/CT [11]. A recent study from our group found that the DMI used herein had 68% higher sensitivity than the PMT-based TOF PET/CT (Discovery 710; GE healthcare) [10]. The major contributor to the sensitivity gain of DMI is the wider axial FOV of 20 cm. Sonni et al. reported that very good quality ^18^F-FDG PET images can be acquired at 90 s/bed using the DMI [17]. The most attractive advantage derived from SiPM-based PET/CT over standard PMT-based PET/CT is more rapid image acquisition for static imaging and is increased SNR in frames of a given duration for dynamic imaging. Because SiPM-based PET/CT decreases acquisition durations as a surrogate for the ^18^F-NaF dose, it might enable reduction of the administered ^18^F-NaF dose while maintaining or improving image quality [17].

The RC increased as a function of decreasing β values as well as with the increasing sphere diameter. The RC is benefited for higher spatial resolution, and Rogasch et al. reported that the spatial resolution of BPL at a lower β value was significantly better than that of OSEM [35]. We considered that the increasing RC is mainly due to the effect of the edge-preserving properties of the relative difference penalty (RDP) in BPL reconstruction at lower β values, which *γ* in RDP is a parameter that controls the degree of edge-preservation [36, 37]. The RC for BPL were superior to those for OSEM, particularly when spheres were smaller. Although OSEM stops after a predetermined number of iterations, resulting in an underconverged image, BPL can reach full convergence of image accuracy without sacrificing image noise [36, 38]. These characteristics of the BPL are considered to increase quantitative accuracy. In the same context, the RC linearity over a range of activity was better for BPL than for OSEM, which was independent of acquisition duration (Fig. 6). Compared with OSEM, BPL might be able to maintain the ability to quantitatively detect potential bone metastases in ^18^F-NaF PET/CT images acquired over shorter durations.

The noise characteristics and vertebral SNR in BPL were superior to those in OSEM (Fig. 8). Thus, the SUV_max_ values were lower in BPL than in OSEM. Win et al. acquired ^18^F-NaF PET images in TOF mode for 3 min/bed [21]. They showed that the average SUV_max_ in normal thoracic vertebrae was 7.36 (range 6.99–7.66). Our average SUV_max_ of 7.71 in BPL reconstruction at 90 s/bed was similar to their findings. The BPL becomes smoother and less noisy depending on β values. Thus, BPL improved the SNR in the background region more effectively than OSEM and was consistent with prior findings using liver SNR [39, 40]. The better image quality of BPL with a β value of 400 in the clinical study compared with OSEM, which was compatible with the findings of our custom-designed phantom. However, we identified variability of the SUV, CV, and SNR among patients with a normal spine. We considered that this resulted from a difference not only in bone metabolism among patients, but also in the physique of patients. Such variability should be expected when images are acquired from overweight patients for whom reducing the acquisition duration is usually not advised.

The present study is limited by the fact that data were generated using a phantom simulation of a clinical exam of an average-sized Japanese patient. Further studies should investigate clinical PET images of patients over a variety of patient habitus. Prolonged acquisitions are generally considered important for improving the quality of PET images acquired from overweight patients. Chilcott et al. reported that image quality is better using BPL, than OSEM reconstruction, with the greatest benefit being for the heaviest of patients [41]. Further examinations of such patients might further demonstrate the advantages of the SiPM-based PET/CT scanner with the BPL algorithm.

## Conclusion

The present study determined optimal parameters for BPL reconstruction with which ^18^F-NaF PET/CT images acquired over different durations. Our results suggested that the high quality and quantitative accuracy of images acquired during shorter durations (90 s/bed) can be maintained better by BPL (β = 400) than by OSEM. The information obtained from the custom-designed phantom study clarifies that SiPM-based PET/CT scanners with BPL reconstruction can detect potential bone metastases in ^18^F-NaF PET/CT images.

## Data Availability

The datasets used and/or analyzed during the current study are available from the corresponding author on reasonable request.

## Abbreviations

PET/CT: Positron emission tomography/computed tomography
^18^F-NaF: ^18^F-sodium fluoride
SPECT: Single-photon emission computed tomography
SUV: Standardized uptake values
SNMMI: Society of Nuclear Medicine and Molecular Imaging
EANM: European Association of Nuclear Medicine
^18^F-FDG: ^18^F-fluoro-2-deoxy-D-glucose
BPL: Bayesian penalized likelihood reconstruction
PSF: Point spread function
OSEM: Ordered-subset expectation maximization
SNR: Signal-to-noise ratio
SiPM: Silicon photomultiplier
PMT: Photomultiplier tubes
TOF: Time-of-flight
NECR: Noise equivalent count rate
FOV: Fields of view
FWHM: Full width at half maximum
AC: Activity concentration
TNR: Tumor-to-normal bone ratio
3D: Three-dimensional
ROI: Regions of interest
CV: Coefficients of variance
RC: Recovery coefficients
SD: Standard deviation
MIP: Maximum intensity projection
